# Dynamics of cooperative networks associated with gender among South Indian Tamils

**DOI:** 10.1098/rstb.2021.0437

**Published:** 2023-01-16

**Authors:** Cohen R. Simpson, Eleanor A. Power

**Affiliations:** ^1^ Nuffield College, University of Oxford, Oxford OX1 1NF, UK; ^2^ Department of Methodology, The London School of Economics and Political Science, London WC2A 2AE, UK; ^3^ The Santa Fe Institute, Santa Fe, NM 87501, USA

**Keywords:** social networks, cooperation, social support, stochastic actor-oriented models, complex systems, India

## Abstract

Helping behaviour is thought to play a major role in the evolution of group-living animals. Yet, it is unclear to what extent human males and human females use the same strategies to secure support. Accordingly, we investigate help-seeking over a 5-year period in relation to gender using data from virtually all adults in two Tamil villages (*N* = 782). Simulations of network dynamics (i.e. stochastic actor-oriented models) calibrated to these data broadly indicate that women are more inclined than men to create and maintain supportive bonds via multiple mechanisms of cooperation (e.g. reciprocity, kin bias, friend bias, generalized exchange). However, gender-related differences in the simulated dynamics of help-seeking are modest, vary based on structural position (e.g. out-degree), and do not appear to translate to divergence in the observed structure of respondents' egocentric networks. Findings ultimately suggest that men and women in the two villages are similarly social but channel their sociality differently.

This article is part of the theme issue ‘Cooperation among women: evolutionary and cross-cultural perspectives’.

## 1. Introduction

Social support—i.e. subtle dyadic cooperation whereby one actor helps another at some cost [1]—is thought to play a major role in the evolution of human and non-human animals. Supportive social bonds are believed to buffer resource shortfalls [2,3], regulate stress [4,5], modulate disease risk [6], bolster longevity [4,6,7], and enhance offspring survival [3,8–10]. Furthermore, these connections are believed to enable members of our species—as well as rather different animals such as lemurs and wasps—to engage in social learning [11–15]. Thus, clarifying the determinants of helping behaviour is a major scholarly task. And evolutionary scientists are especially interested in understanding how biological sex shapes supportive action (e.g. food provision, knowledge sharing, and grooming).

As Mattison *et al*. [16] discuss, evolutionary theorizing suggests that the social ties (i.e. non-sexual social relationships) of human males and human females will differ due to their pursuit of distinct strategies for reproduction, economic production, and cooperation (see also [2,17–23]). Females are thought to be as social as males [18] and equally capable of making net-positive economic contributions [20,21,24]. However, females are expected to prioritize establishing social ties conducive to childcare. This is due to the physical and knowledge investments required throughout gestation, nursing, and parenting during the long period of childhood [2,10,20,25,26], in addition to the challenges of variable resource access and variable male presence [2,19,26]. By contrast, males are expected to prioritize establishing social ties conducive to achieving status [18,27,28] and easing male–male collective action (i.e. group cooperation such as hunting and war [2,29,30])—particularly in the wake of conflict [30].

**Figure 1.**
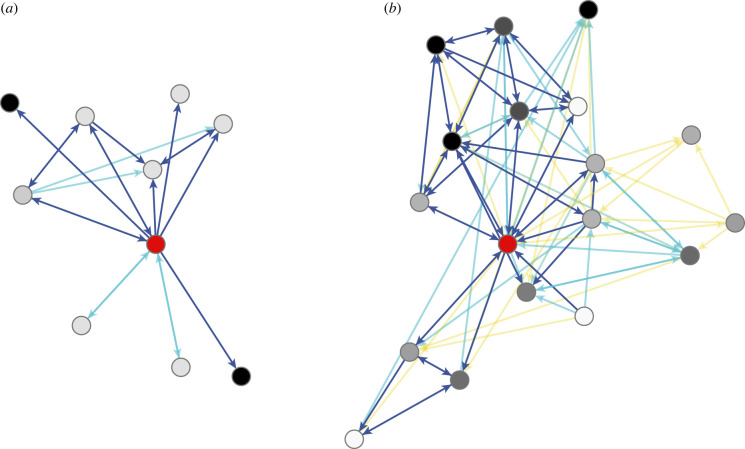
Stylization of sex-homogeneous one-degree (i.e. one-step) egocentric networks. Stylization is based on evolutionary theorizing of male and female sociality as, respectively, ‘dyadic’ and ‘group-based’ [17,18,23]. Arcs (i.e. directed connections) indicate hypothetical aid relationships—i.e. to whom does one turn for help?—and are coloured based on relationship type. Dark-blue arcs emanate from kin, light-blue arcs emanate from friends, and yellow arcs emanate from in-group 'strangers' (i.e. associates who are neither kin nor friend). Red vertices (i.e. nodes) indicate ego. Vertices for ego's alters are coloured to reflect social status relative to ego—where darker-coloured vertices are more high-status than ego (i.e. the focal actor), white vertices are more low-status, and grey vertices are of a similar status. (*a*) Theorized female egocentric network characterized by low absolute size, low interconnectivity, a large degree of status homogeneity and no supportive bonds with non-kin and non-friends. (*b*) Theorized male egocentric network characterized by large absolute size, high interconnectivity, a large degree of status heterogeneity and multiple supportive bonds between kin, friends, non-kin and non-friends. Lengths of arcs, placement of vertices and spacing between arcs and vertices are purely aesthetic.

With respect to the *structure* (i.e. the arrangement) of social ties, this evolutionary logic yields two broad predictions [16] reflective of several presumed behavioural tendencies on the part of males and females. First, the egocentric (i.e. personal/local/immediate) networks of females are predicted to be smaller than the egocentric networks of males. This is due to females' expected emphasis on intimacy leading to a preference for a restricted set of direct, high-quality ties with known individuals of a similar status, typically kin and friends [17,18,23,31] (figure 1*a*). Second, males are predicted to amass a broad sphere of interconnected social ties and thus build complex (i.e. polyadic) networks. This is due to males’ expected willingness to forgo investment in their conjugal partners to instead pursue hierarchically-organized groups of varying sizes composed of connections of varying quality between friends, kin, and unrelated in-group acquaintances of differing social statuses [16–19,23,32] (figure 1*b*). By contrast, female ego-nets are expected to exhibit low interconnectivity, which, alongside their low size, is thought to make them appear ‘dyadic’ or ‘parochial’ in nature [17,18,23].

These predications are supported by studies of some species of non-human primates, humans in subsistence societies, and humans in advanced economies (e.g. see [17,19,20,23,33–36] but cf. [16,31,35,37–39]). Furthermore, findings from cognate research on the psychology of WEIRD [40] humans [18,34,37,41–44], the psychology of non-human animals [45], and the behaviour of humans on the Internet [46,47] support the idea that the structure of social ties can meaningfully differ between the two sexes. Nevertheless, evolutionary research on adult humans—the focus of this paper—has narrowly focused on comparing the number of supportive relationships that males and females have.

A growing body of evidence does suggest that one's number of social ties is positively associated with fitness in a variety of species, including humans [3,6,8,43,48–50]. However, such counts alone tell us little about how supportive relationships emerge and persist in relation to the population-spanning social networks within which individuals are embedded (e.g. networks of social ties within and across hunter–gather camps [35,51]). Of course, human evolutionary scientists have long been interested in descriptors of structural position more intricate than ego-net size (e.g. eigenvector centrality [39,48,50], betweenness centrality [16,50], Page-rank centrality [52], and closeness centrality [5,50]). Still, analyses of biological sex in relation to positional metrics, regardless of their complexity, are poor vehicles for investigating differences in the social worlds of males and females.

This is not merely pedantic methodological critique. Like friendship [43], supportive social ties impose some costs on individuals [53]. And accumulation of these relationships may trade off against one's own wellbeing (i.e. negative social capital [54]) via psychological discomfort [53], burdensome indebtedness [54,55], and resource depletion [3,54] even if social support affords immediate fitness-relevant benefits beyond help itself [3,5,50]. Consequently, we can expect both males and females to prefer supportive ties with certain individuals (i.e. certain ‘alters’) over others. Thus, it is important to understand the *process* by which males and females select between alters when presented with an expansive set of possible sources and targets of aid. For example, out of all other residents in a female's village, who will she turn to for help? And how might the patterning of help-seeking across her entire village mould her choice? Analyses of positional metrics are ill suited to answer such questions as they do not consider how males and females choose between cooperative relationships with different cost–benefit profiles.

Furthermore, positional metrics are themselves functions of the structure of population-spanning social support networks. These global social structures emerge from, provide context for, and are continuously remade by individuals' decisions to build—or to not build—cooperative ties with specific alters (see [56–61] on the 'micro-macro link'). Thus, positional metrics can only be used to indirectly test the two evolutionary predictions above. This is because both predictions are, in the case of social support, multidimensional conjectures about how males and females are expected to choose their sources and targets of aid—where such choice precedes ego position.

Accordingly, here we test the two evolutionary hypotheses about the structure of males' and females’ social ties with sociocentric (i.e. whole network) analysis. Specifically, we build individual-oriented simulation models of the temporal evolution of social support over a 5-year period in two adjacent, patrilocal and patrilineal villages in Tamil Nadu, India. As the two evolutionary predictions outlined above collapse distinct mechanisms of cooperation [62], we disaggregate them into their constituent presuppositions, hypothesizing that:

**H1**: Females are less inclined to create and maintain supportive bonds in general.
**H2**: Females are more inclined to create and maintain supportive bonds embedded within reciprocated dyads.
**H3**: Females are more inclined to create and maintain supportive bonds with kin.
**H4**: Females are more inclined to create and maintain supportive bonds with friends.
**H5**: Females are less inclined to create and maintain supportive bonds embedded within polyadic groups.
**H6**: Females are more inclined to create and maintain supportive bonds with same-status peers.

Owing to our sociocentric approach, we simultaneously analyse ties within and between the two sexes (cf. Redhead & von Rueden [27,28]). Accordingly, H1–H6 are agnostic as to whether supportive bonds are with same- or opposite-sex cooperative partners. That is, our hypotheses do not postulate, in the sense of our individual-oriented regression modelling (discussed below), multiplicative interactions between the sex of ego, the sex of alter, and a specific cooperative mechanism (e.g. *ego*_female_ × *alter*_female_ × reciprocity), only between the sex of ego and a specific mechanism (e.g. *ego*_female_ × reciprocity). However, some evolutionary scientists distinguish cooperation within and between the sexes (see Jaeggi *et al*. [21], Redhead & von Rueden [27,28], and Wrangham & Benenson [23]). And while males and females are both subject to homophily (i.e. assortative mixing), males are expected to exhibit a greater preference for same-sex ties compared to females whose desire for same-sex relationships is thought to be primarily satisfied via kin and friends [17,23]. Consequently, we also hypothesize that:

**H7**: Females are less inclined to create and maintain supportive bonds with same-sex peers.

## 2. Methods

### (a) Data summary

The data from India were collected during ethnographic fieldwork by the second author E.A.P. [52,63–65] in the pseudonymous villages ‘Tenpaṭṭi’ and ‘Alakāpuram’. Both villages are located near the Vaigai River in Tamil Nadu. Sitting off a major road catering to diverse traffic, the two villages are large and adjacent. Indeed, Tenpṭṭi and Alakāpuram are separated by just 2 km of agricultural fields and scrublands. Subsistence from agriculture (i.e. rice, cotton, sugarcane, and vegetables) is mostly limited to a few months during the year. And as a result, wage labour (e.g. construction and wood cutting) is prevalent. Still, young residents typically complete secondary school and aim to pursue diploma courses, bachelor's degrees, and semiskilled jobs in factories, shops, and offices in nearby towns. Since the mid-twentieth century, fertility rates in Tamil Nadu have fallen, with women having fewer children over shorter reproductive periods, due to, for example, delayed first birth, delayed marriage, and the rise of family planning [63]. Post-marital residence is generally patrilocal. However, there are a sizeable number of women who continue to reside in their natal village, even after marriage [63]. Both villages comprise a mix of religious (Hindu and Christian) and caste groups. These castes are largely Scheduled-class (relatively deprived) and Backward-class (relatively better-off), in the terminology of the Indian government. A larger share of Alakāpuram's residents are Scheduled Caste.

Two waves of sociometric data were collected in February/April 2013 and in September 2017. Virtually all adult (18+) residents of Tenpaṭṭi and Alakāpuram (i.e. 782/809 or 97% in 2013 and 788/817 or 96% in 2017) were asked a series of sociometric questions concerning who they turn to for different types of support, as well as who they saw as having a range of desirable reputational qualities. For this study, we focus on named sources of: (i) advice; (ii) wage labour; (iii) quotidian physical assistance (e.g. household chores); (iv) essential household consumables (e.g. rice, sugar, oil); (v) money; and (vi) general conversation.

Ancillary socio-demographic data come from a household census also carried out by E.A.P. In addition to detailing gender identity—which we expect to rarely deviate from biological sex given the setting—these data cover a range of geographic, demographic, and economic factors likely to constrain the interactions of men and women. These factors include kinship relations, distance between households, household membership, age, years of education, household wealth (Indian Rupees [*INR*]), caste, village immigration status (i.e. non-natal village resident versus natal village resident), and partnership status (i.e. not married versus married). Using the sociometric data, we also construct a measure of general reputation (i.e. the sum of one's intra-village nominations as ‘generous’, ‘influential’, of a ‘good character’, and ‘strong’).

### (b) Network construction

We use villagers’ reports on who they turn to for the six types of support above to construct two networks—one for each wave *m* ∈ {2013, 2017}—that span Tenpaṭṭi **and** Alakāpuram. In other words, we have two binary, asymmetric adjacency matrices *x* that encode, across both villages, composite aid relationships—i.e. *to whom does one turn to for one or more kinds of help?* Formally, *x_ij_*(*t_m_*) = 1 if villager *i* seeks one or more kinds of aid from villager *j* according to *i* at wave *m*—where *i* and *j* may, or may not be, in the same village. In total, there are 5362 composite asymmetric aid relationships in the 2013 adjacency matrix *x*(*t*_2013_) and 4085 in the 2017 adjacency matrix *x*(*t*_2017_).

Sociometric questions used to elicit villagers' sources of aid in 2013 and 2017 appear in the electronic supplementary material in table S1 (see also electronic supplementary material, *Network Measurement*). In electronic supplementary material, *Network Composition Change*, we discuss turnover in the populations of Tenpaṭṭi and Alakāpuram between the two waves and how we handle this in our statistical models. Descriptive statistics for the 2013 and 2017 adjacency matrices appear in the electronic supplementary material in table S2 and table S3.

### (c) Stochastic actor-oriented models

To analyse the 2013 and 2017 adjacency matrices, we rely on stochastic actor-oriented models (SAOMs) [59,66–71]. These models are used for observational analyses of the temporal evolution of networks. Put simply, SAOMs are akin to multinomial logistic regression. More formally, and as previously summarized by the first author C.R.S. [62], SAOMs are simulations of individual network members’ choices between **outgoing** relationships with different rewards and costs. SAOM simulations are calibrated or ‘tuned’ to discrete observations of a network. That is, conditional on the ‘snapshots’ of a dynamic graph—here, *x*(*t*_2013_) → *x*(*t*_2017_)—SAOMs simulate network evolution between successive waves as a continuous-time process of asynchronous and sequential tie changes [59,66–71].

During a SAOM simulation, focal actors *i* (egos) myopically modify just one of their outgoing relationships with some alter *j* in the set of network members *N* (i.e. *j* ∈ *N*, *j* ≠ *i*). The change made by *i* is the change that maximizes a utility or ‘evaluation’ function, where ‘no change’ is also an option. In this respect, the evaluation function captures the ‘attractiveness’ [70] of simulated tie changes—where ‘attraction’ means ‘…something like ‘sending a tie to [an actor *j*] with a higher probability if all other circumstances are equal.' (Snijders & Lomi [72, p. 5]).

**Figure 2.**
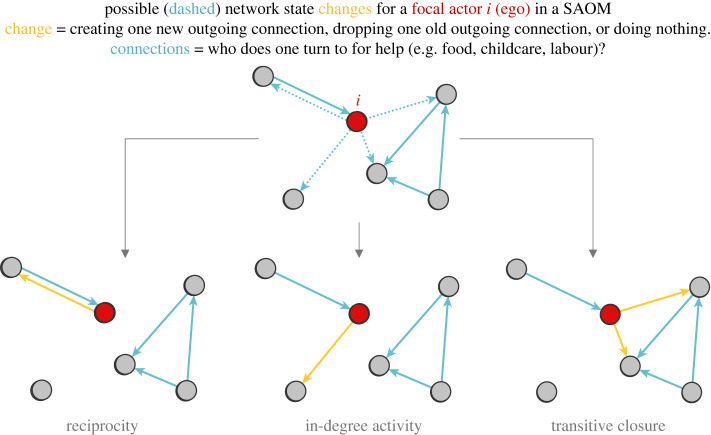
Stylization of ‘change’ in a stochastic actor-oriented model (SAOM) vis-à-vis network-formation mechanisms. Note the two outgoing ties in a transitive triad—both of which are under the control of the focal actor *i* (ego) and only one of which is eligible to be changed at a time. Also, note that mechanisms are not always mutually exclusive. For example, transitive closure is related to popularity bias (n.b., the incoming ties of the actors with whom *i* can connect). We are grateful to anonymous reviewer 2 for proposing this schematic.

The evaluation function is a weighted sum of parameter estimates $\hat{\beta }$ and their associated covariates *k* (i.e. SAOM ‘effects’ [70] plus an additional variable used to capture random influences [69]. The simulated tie changes or ‘ministeps’ [70] made by *i* shift the network between adjacent (unobserved) states. These states differ, at most, by the presence/absence of a single tie [59,71] (e.g. figure 2). The probabilities of the ministeps—a large number of which are required to bring one discrete observation of the network to the next (i.e. *x*(*t*_2013_) → *x*(*t*_2017_))—are given by a multinomial logit which uses the evaluation function as the linear predictor.

Each covariate *k* used to specify the evaluation function summarizes some structural (i.e. purely network-related) feature or non-structural feature of *i*'s immediate (i.e. local) network. Examples include the sum of the in-degrees of *i*'s alters, the number of reciprocated dyads that *i* is embedded in, or *i*'s number of outgoing ties weighted by genetic relatedness. These features correspond to theoretical mechanisms of interest (e.g. preferential attachment, reciprocal altruism, or kin selection).

### (d) Interpretation of stochastic actor-oriented models

SAOM parameter estimates $\hat{\beta }$ (log odds ratios) summarize the association between the covariates and the *simulated* tie changes or ‘ministeps’. Specifically, let *i* indicate some focal network member. Let *x* represent some current (i.e. status-quo) network state. And let *x*^±^*^ij^* represent the network that is *adjacent* to *x* such that it is defined by *i*'s addition/subtraction of the tie *x_ij_* to/from *x*. Given the opportunity to make a ministep in departure from *x* to *x^±ij^*, ${\hat{\beta }}_{k}$ is the log odds of *i* choosing between two different versions of *x*^±^*^ij^* in relation to some covariate *k*. For example, ${\hat{\beta }}_{\mathrm{Reciprocity}}=1.7$ would indicate that the log odds of *i* creating and maintaining the supportive relation *x_ij_* is, conditional on the other covariates *k*, larger by 1.7 when *x_ij_* reciprocates a tie (i.e. *x_ji_*) compared to when *x_ij_* does not reciprocate a tie (i.e. reciprocated ties are more ‘attractive’).

Given the longitudinal nature of the model, the gain in the evaluation function for a ministep is determined by the *difference* Δ between the value of the statistic *s* for a covariate *k* induced by *i*'s addition/subtraction of *x_ij_* to/from *x*—i.e. Δ*_k_*_,*ij*_(*x*, *x*^±^*^ij^*) = *s_k_*_,*i*_(*x*^±*ij*^) − *s_k_*_,*i*_(*x*). These differences are known as ‘change statistics’ (see Block *et al*. [71] and Ripley *et al*. [70]). And ${\hat{\beta }}_{\mathrm{Reciprocity}}=1.7$, for example, is the value that *x_ij_* positively contributes to the evaluation function when *x_ij_* increases the network statistic *s_k_*_,*i*_(*x*) for the *Reciprocity* effect by the value of one (i.e. Δ_Reciprocity,*ij*_(*x*, *x*^±^*^ij^*) = *s*_Reciprocity,*i*_(*x*^±^*^ij^*) − *s*_Reciprocity,*i*_(*x*) = 1 − 0 = 1).

### (e) Model specification

For our analysis, we fit three SAOMs using nested specifications. The first SAOM (i.e. Model 1) is our baseline specification. It features a parsimonious set of effects reflective of (i) the evolutionary theories of cooperation undergirding our hypotheses (e.g. kin selection and reciprocal altruism); as well as (ii) sociologists' understanding of interdependence between positive-valence (i.e. not based on disliking or aggression), asymmetric relationships [59,61,71,73] (e.g. transitive closure and popularity-bias). In the second SAOM (i.e. Model 2), we add multiplicative interactions between gender (1 = woman; 0 = man) and all effects in the baseline specification (e.g. kinship, reciprocity, transitivity) to allow network dynamics to unfold differently between men and women [74]. Last, in our third SAOM (i.e. Model 3), we expand the specification used for Model 2 with variables such as age, wealth, and immigration status. This adjustment reflects the sociological argument that observed sex- and gender-based differences in network structure are not due to sex and gender *per se* but are instead the result of sex and gender simply being correlated with factors that constrain sociality [31,38,53,75–80] (see also electronic supplementary material, *Sociological Determinants of Social Ties*).

**Figure 3.**
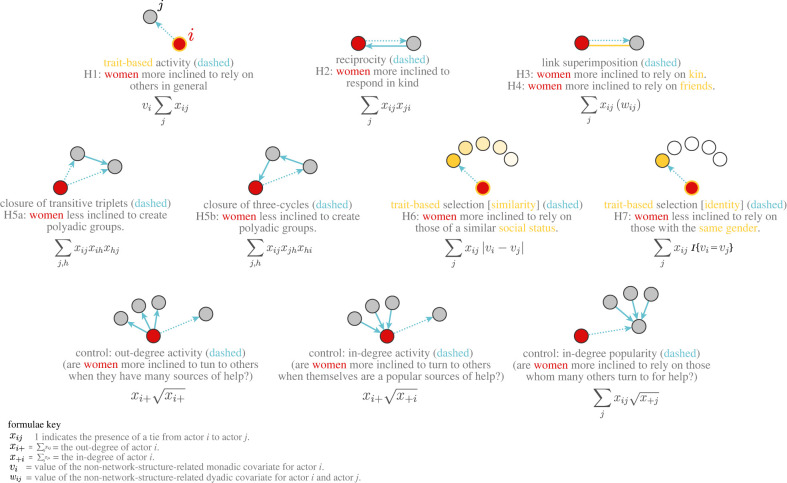
Network dynamics investigated with stochastic actor-oriented models (SAOMs) vis-à-vis hypotheses alongside key network-structure-related controls. Formulae are used to calculate the network statistics *s_k_*_,*i*_(*x*)—i.e. the covariates in our SAOMs (see also electronic supplementary material, table S6). Network statistics generally take the form of actor-specific counts over all network members *j* to which some focal actor *i* is tied. Note the two outgoing ties in a transitive triad—both of which are under the control of *i* and only one of which is eligible to be changed at a time. We are grateful to anonymous reviewer 2 for proposing this schematic.

Figure 3 visualizes the SAOM effects related to our hypothesized theoretical mechanisms. In the electronic supplementary material, we explain the settings used to estimate our SAOMs, how we specify our baseline SAOM vis-à-vis prior research and goodness-of fit, and why we prefer interactions with gender. Descriptive statistics for our monadic and dyadic covariates appear in the electronic supplementary material in tables S4 and S5. And table S6 in the electronic supplementary material provides the formulae used to calculate the network statistic *s_k_*_,*i*_(*x*) underpinning each constitutive (i.e. ‘main’) effect *k* used to specify our SAOMs alongside short verbal descriptions.

### (f) Assessment of gender-based differences using linear combinations

To test our hypotheses, we follow the architects of the SAOM [70,72,81] by using artificial scenarios for *i*'s creation and maintenance of a single outgoing tie *x_ij_*. Specifically, and in line with Brambor *et al*.'s [82] best practices for interpreting multiplicative interactions, we gauge the evidence in favour of each of our predictions using linear combinations (i.e. weighted sums) of parameter estimates ${\hat{\beta }}_{k}$ from our fully specified SAOM (i.e. Model 3; electronic supplementary material, table S7) and synthetic change statistics Δ_*k*,*ij*_. We create the change statistics using plausible, but artificial values for network statistics *s_k_*_,*i*_ that represent a status-quo network state *x* to which a hypothetical tie *x_ij_* is added. This allows us to calculate the total contribution to the evaluation function—i.e. the overall ‘attractiveness’ [70] of creating and maintaining a single hypothetical tie *x_ij_*—for the set of effects associated with each of our hypotheses.

In the electronic supplementary material in table S8, we provide the eight hypothesis-specific linear combinations of parameter estimates from Model 3. Each linear combination includes the interaction effect of interest (e.g. ${\hat{\beta }}_{\mathrm{Woman}\;\times \;\mathrm{Reciprocity}}$) in addition to the constitutive (i.e. ‘main’) effects (e.g. ${\hat{\beta }}_{\mathrm{Woman}}$ and ${\hat{\beta }}_{\mathrm{Reciprocity}}$) and any other effects necessarily implicated in the creation and maintenance of a single tie *x_ij_* (e.g. ${\hat{\beta }}_{\mathrm{Out}-\mathrm{degree}\;\mathrm{Activity}}$, ${\hat{\beta }}_{\mathrm{In}-\mathrm{degree}\;\mathrm{Popularity}}$ and ${\hat{\beta }}_{\mathrm{In}-\mathrm{degree}\;\mathrm{Activity}}$; figure 3). For our linear combinations, we limit our attention to a focal actor (*i*; ego) who is either a woman or a man with otherwise identical characteristics and identical structural positions—where *i* targets patrons (*j*; alters) who are themselves identical. Owing to space constraints, we provide critical, additional detail on how we build the linear combinations with respect out-degree, in-degree, reciprocity, transitive closure and cyclic closure in electronic supplementary material, *Set-Up of Linear Combinations*. However, note that we generally use combinations of the observed ranges and the median values of network statistics in our 2013 data to construct our linear combinations.

Last, we stress two points. First, our linear combinations relate to the log odds of *x_ij_*—not the probability of *x_ij_* and, given the size of our network, the probability of any one tie is expected to be small. Second, SAOMs model network evolution from the perspective of ego (hence ‘actor-oriented’), where effects in SAOMs are generally functions of one another. Practically speaking, this makes several components of the linear predictor interdependent—not only those components that are involved in a multiplicative interaction as typically encountered in standard regression models (e.g. ${\hat{\beta }}_{\mathrm{Woman}\;\times \;\mathrm{Reciprocity}}$, ${\hat{\beta }}_{\mathrm{Woman}}$, and ${\hat{\beta }}_{\mathrm{Reciprocity}}$; see again electronic supplementary material, table S8). Thus, it is inadvisable to speak of any one effect alone.

### (g) Data transformations

We fit our SAOMs using a series of binary dyadic indicators (e.g. *Kinship*, *Friendship, Same Gender, Same Caste*) and standardized scores (i.e. *Z*-scores) of age, household wealth (i.e. Log*_e_ INR*), social standing (i.e. $\sqrt{\mathrm{General}\;\mathrm{Reputation}}$), and pairwise geographic distance (i.e. Log*_e_* metres + 1) by subtracting from each variable its global mean across the two villages and then dividing by its global standard deviation. Furthermore, we adjust for heterophily using the absolute value of the differences between villagers' quantitative attributes (i.e. age, village [Tenpaṭṭi = 1, Alakāpuram = 0], and $\sqrt{\mathrm{General}\;\mathrm{Reputation}}$). See also electronic supplementary material, *Interpretation of SAOM Results Given Data Centring*.

## 3. Results

**Figure 4.**
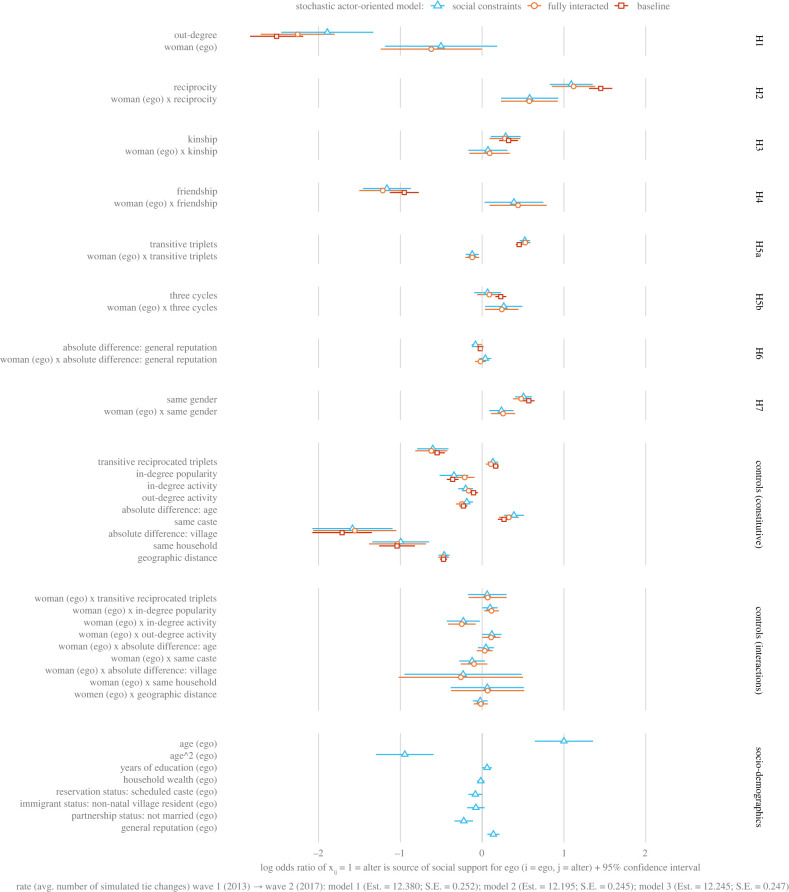
Parameter estimates $\hat{\beta }$ from the three stochastic actor-oriented models (SAOMs) of social support within and across Tenpaṭṭi and Alakāpuram. SAOMs fit using standardized scores (i.e. *Z*-scores) of age (mean = 44.01; s.d. = 14.70), household wealth (i.e. Log*_e_ INR*; mean = 12.62; s.d. = 0.94), social standing (i.e. $\sqrt{\mathrm{General}\;\mathrm{Reputation}}$; mean = 2.28; s.d. = 1.97), and pairwise geographic distance (i.e. Log*_e_* metres + 1; mean = 5.69; s.d. = 1.36). Log- and square-root transformations taken prior to standardization. s.d. = standard deviation.

**Figure 5.**
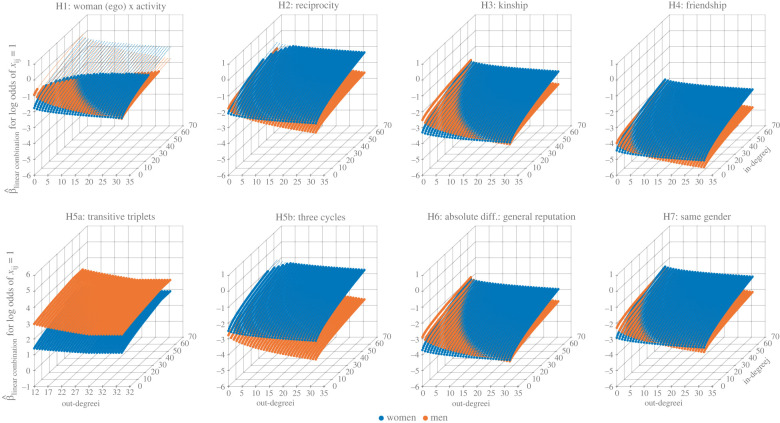
Gender-based differences in the simulated micro-level dynamics of help seeking. Each hypothesis-specific linear combination ${\hat{\beta }}_{\mathrm{Linear}\;\mathrm{Combination}}$ denotes the total contribution to the evaluation function—i.e. the overall ‘attractiveness’ [70] of creating and maintaining a single outgoing tie *x_ij_*—for a focal actor *i* (ego) who is either a woman (blue bullets) or man (orange bullets) with identical characteristics (e.g. average age and average wealth) and identical structural positions. Each bullet is a linear combination. The linear combinations themselves are sums of key parameter estimates ${\hat{\beta }}_{k}$ from Model 3 (Social Constraints) and artificial change statistics Δ_*k*,*ij*_(*x*, *x*^±^*^ij^*) (see electronic supplementary material, table S8). The change statistics indicate the *difference* in network statistics *s_k_*_,*i*_ (electronic supplementary material, table S6) representative of some status-quo network state *x* and some new network state *x*^±^*^ij^* induced by *i*'s addition/subtraction of a single tie *x_ij_* to/from *x*. Linear combinations represent artificial status quo networks *x* wherein *i*'s out-degree varies from zero to 32, *i*'s in-degree is fixed at six, the in-degree of those to whom *i* is tied is fixed at six, *j*'s in-degree varies from zero to 64, and *j*'s out-degree is ignored. There are 2145 (33 × 65) linear combinations for the average man and the average woman for each cooperative mechanism (H1–H7) or 4290 per sub-plot. Tiny bullets (e.g. H1, top-left) are for linear combinations that have a *p*-value ≥ 0.001—where each *p*-value (two-tailed) is associated with the test statistic $z_{{\hat{\beta }}_{\mathrm{Linear}\;\mathrm{Combination}}}={\hat{\beta }}_{\mathrm{Linear}\;\mathrm{Combination}}÷s.e._{{\hat{\beta }}_{\mathrm{Linear}\;\mathrm{Combination}}}$. The standard error (s.e.) for each linear combination was obtained with the procedure of Ripley *et al*. [70, pp. 95–97]. Using *Reciprocity* as an example, linear combinations generally take the form: ${\hat{\beta }}_{\mathrm{Out}-\mathrm{degree}}\left(\Delta_{\mathrm{Out}-\mathrm{degree},\;ij}\right)+{\hat{\beta }}_{\mathrm{Woman}}\left(\Delta_{\mathrm{Ego}-\mathrm{Activity}:\;\mathrm{Gender},\;ij}\right)+{\hat{\beta }}_{\mathrm{Reciprocity}}\left(\Delta_{\mathrm{Reciprocity},\;ij}\right)+{\hat{\beta }}_{\mathrm{Woman}\;\times \;\mathrm{Reciprocity}}(\Delta_{\mathrm{Ego}-\mathrm{Activity}:\;\mathrm{Gender},\;ij}\times$ $\Delta_{\mathrm{Reciprocity},\;ij})+{\hat{\beta }}_{\mathrm{Out}-\mathrm{degree}\;\mathrm{Activity}}\left(\Delta_{\mathrm{Out}-\mathrm{degree}\;\mathrm{Activity},\;ij}\right)+{\hat{\beta }}_{\mathrm{In}-\mathrm{degree}\;\mathrm{Activity}}\left(\Delta_{\mathrm{In}-\mathrm{degree}\;\mathrm{Activity},\;ij}\right)+{\hat{\beta }}_{\mathrm{In}-\mathrm{degree}\;\mathrm{Popularity}}\left(\Delta_{\mathrm{In}-\mathrm{degree}\;\mathrm{Popularity},\;ij}\right)+{\hat{\beta }}_{\mathrm{Woman}\;\times \;\mathrm{Out}-\mathrm{degree}\;\mathrm{Activity}}$ $\left(\Delta_{\mathrm{Ego}-\mathrm{Activity}:\;\mathrm{Gender},\;ij}\times \Delta_{\mathrm{Out}-\mathrm{degree}\;\mathrm{Activity},\;ij}\right)+{\hat{\beta }}_{\mathrm{Woman}\;\times \;\mathrm{In}-\mathrm{degree}\;\mathrm{Activity}}\left(\Delta_{\mathrm{Ego}-\mathrm{Activity}:\;\mathrm{Gender},\;ij}\times \Delta_{\mathrm{In}-\mathrm{degree}\;\mathrm{Activity},\;ij}\right)+{\hat{\beta }}_{\mathrm{Woman}\;\times \;\mathrm{In}-\mathrm{degree}\;\mathrm{Popularity}}(\Delta_{\mathrm{Ego}-\mathrm{Activity}:\;\mathrm{Gender},\;ij}\times$ $\Delta_{\mathrm{In}-\mathrm{degree}\;\mathrm{Popularity},\;ij})$, where ${\hat{\beta }}_{k}(\Delta_{\mathrm{Reciprocity},ij})={\hat{\beta }}_{\mathrm{Reciprocity}}$ $\left(s_{\mathrm{Reciprocity},i}(x^{\pm \;ij})-s_{\mathrm{Reciprocity},i}(x)\right)$. Note, H6 concerns a one unit increase in the absolute difference between the Z-score of ${\sqrt{\mathrm{General}\;\mathrm{Reputation}}}_{i}$ and ${\sqrt{\mathrm{General}\;\mathrm{Reputation}}}_{j}$ (i.e. Δ_Abs. Diff.: General Rep.,*ij*_(*x*, *x*^±^*^ij^*) = 1 − 0). Thus, the linear combinations for H6 summarize the attractiveness of *x_ij_* for two actors with a one-standard-deviation difference in $\sqrt{\mathrm{General}\;\mathrm{Reputation}}$, where H6 implies higher levels of attractiveness for *men* in relation to this difference.

Figure 4 visualizes the parameter estimates $\hat{\beta }$ from our SAOMs of social support (Model 1 [Baseline], Model 2 [Fully Interacted], Model 3 [Social Constraints]). Figure 5 visualizes the hypothesis-specific linear combinations of estimates from Model 3 using different combinations of out-degree for ego (*i*) and in-degree for alter (*j*).

Given how we transformed our data to fit our SAOMs (see above), in conjunction with how we have selected the terms to include in our linear combinations, our results relate to the global ‘typical’ dyad in the two villages. Specifically, our linear combinations summarize the attractiveness of creating and maintaining a supportive bond *x_ij_* between two identically aged network members *i* and *j* who are from different castes, who live 298.86 metres (*e*^5.7^) apart in the same village, and who may or may not be directly connected (H2), kin (H3), friends (H4), indirectly connected (H5), the same social status (H6), or the same gender (H7) depending on the linear combination (electronic supplementary material, table S8). Furthermore, given the actor covariates in Model 3, *j* only differs based on their in-degree and *i* always has ‘typical’ features (i.e. a man/woman who is a married, Backward-caste, natal-village resident of average age, education, wealth, and social standing). Recall the additive nature of the SAOM evaluation function in its basic form, where we isolate the effect of gender-based homophily (i.e. *Same Gender* = 1 versus *Same Gende*r = 0) to test H7 (electronic supplementary material, table S8). Thus, note in particular that results for H1-H6 relate to dyads wherein *i* and *j* have *different* genders.

Also note that it is not surprising that most linear combinations are negative—indicating that, conditional on the other effects in the model, those effects involved in the linear combination *by themselves* (electronic supplementary material, table S8) make it less likely to create and maintain a supportive relationship. To clarify, the network spanning Tenpaṭṭi and Alakāpuram is very sparse, the linear combinations generally ignore clustering and homophily, and all combinations include the *Out-degree* effect. Akin to an intercept, the *Out-degree* effect reflects the basic tendency to have ties at all [81]. And, under a decision-theoretic interpretation of the SAOM, its negative value (figure 4) indicates that the benefits of creating and maintaining a supportive bond *x_ij_* with an arbitrary alter *j* fail to outweigh the costs [81]. Accordingly, what is most important here is the relative attractiveness of a tie for men and women.

### (a) Do men and women diverge in their strategies for accessing aid?

There is compelling evidence (*p* value < 0.001) to suggest that the attractiveness of creating and maintaining a supportive bond *x_ij_* in general (H1) is higher for men (orange bullets, figure 5) with lower out-degrees, specifically when *x_ij_* targets (as our ‘typical’ dyad implies) an identically aged within-village woman (n.b., *Same Gender* = 0) with fewer incoming ties and with whom *i* has the same social standing but no third-party connections, no direct connection (i.e. *x_ji_* = 0), no familial or friendly bond, and no shared caste identity. By contrast, the attractiveness of *x_ij_* in this scenario is higher for women (blue bullets, figure 5) who have larger out-degrees, particularly when *x_ij_* targets a man with many incoming ties.

This ‘cross-over’ in the attractiveness of *x_ij_* for men and women within a typical dyad as the out-degree of *i* and the in-degree of *j* grow also characterizes the attractiveness of *x_ij_* vis-à-vis kinship (H3), reputation-based disassortativity (H6; see end of caption for figure 1), gender-based assortativity (H7), and, to a much-lesser degree, reciprocity (H2) and friendship (H4). This cross-over represents mixed evidence in relation to H1, H2, H3, H4, H6, and H7. However, *x_ij_* appears to be more attractive to women in a typical dyad in relation to reciprocity and friendship across the great majority of the linear combinations where *p* < 0.001. This is consistent with H2 and H4, respectively.

As for polyadic groups, there is clear gender-based divergence in the attractiveness of creating and maintaining a supportive bond *x_ij_* under transitive closure (H5a) and cyclic closure (H5b) for the typical dyad. For H5a, linear combinations concern the tie *i* → *j* in the transitive triad [*i* → *h* → *j* ← *i*], not *i* → *h*. And for H5b, the linear combinations concern the tie *i* → *j* in the cyclic triad [*i* ← *h* ← *j* ← *i*]. The attractiveness of a cross-gender supportive bond *x_ij_* is substantially higher for a man compared to an otherwise-similar woman when *x_ij_* closes a median number of outbound two-paths [*i* → *h* → *j*] (i.e. 12) and targets an identically aged village coresident with whom *i* has the same social standing but no direct connection, no familial or friendly bond, and no shared caste identity. By contrast, the attractiveness of *x_ij_* in this scenario is higher for a woman compared to an otherwise-similar man when *x_ij_* closes a median number of in-bound two-paths [*i* ← *h* ← *j*] (i.e. 5). The gender-based difference in the attractiveness of *x_ij_* under transitive closure is consistent with H5, although it narrows somewhat as *i*'s out-degree increases. However, the difference in the attractiveness of *x_ij_* under cyclic closure conflicts with H5. And it becomes starker as *i*'s out-degree increases.

Last, it is important to consider our findings around specific cooperative mechanisms in relation to each other. To assess the amount of evidence for gender-based differences in the operation of distinct cooperative dynamics [62] (e.g. transitivity, homophily and kin bias), our linear combinations (electronic supplementary material, table S8) do not ‘mix’ hypothesized interactions (e.g. ${\hat{\beta }}_{\mathrm{Woman}\;\times \;\mathrm{Transitive}\;\mathrm{Triplets}}$ plus ${\hat{\beta }}_{\mathrm{Woman}\;\times \;\mathrm{Same}\;\mathrm{Gender}}$) and their constitutive effects (e.g. ${\hat{\beta }}_{\mathrm{Transitive}\;\mathrm{Triplets}}$ plus ${\hat{\beta }}_{\;\mathrm{Same}\;\mathrm{Gender}}$). However, given well-documented tendencies for triangulation in human social networks, for example, this can make our scenarios for a ‘typical’ dyad rather *atypical*. This is because it is very likely that a single tie *x_ij_* with, for instance, a friend, a relative, or a same-gender peer stands to close one or more out-bound two-paths [*i* → *h* → *j*]. Thus, transitivity and, say, gender-based homophily could simultaneously determine the attractiveness of a tie by contributing to the SAOM's evaluation function in an additive manner (cf. a multiplicative contribution via an interaction between *Transitive Triplets* and *Same Gender*). Practically speaking, this means that both the direction and the divergence in the attractiveness of *x_ij_* between men and women under transitivity could characterized the attractiveness of *x_ij_* in relation to the other cooperative mechanisms considered here depending on the non-network-related traits and the structural positions of ego and alter. *Mutatis mutandis* for the effects *Three Cycles*, *Reciprocity*, *Kinship*, and *Friendship*. This all underscores the contrived nature of our scenarios for the linear combinations and the great difficulty of interpreting effects in statistical models of the formation of complex networks such as the SAOM.

### (b) Do gender-based differences in strategies for accessing aid translate to men's and women's ego-networks?

Given prior research by human evolutionary scientists [5,16,39,48,50,52], it is useful to consider our models in relation to differences in the overall structure of the egocentric networks of men and women in Tenpaṭṭi and Alakāpuram. Accordingly, we use the SAOM goodness-of-fit tests of Lospinoso & Snijders [83] to compare aspects of the egocentric networks of men and women. This comparison concerns ego-nets from our 2017 adjacency matrix and 10 000 synthetic versions of this matrix simulated under each fitted model (table 1). This comparison reflects all effects used to specify each of our SAOMs (figure 4).

**Table 1.** Wald tests and goodness of fit (GOF) for stochastic actor-oriented models of composite social support among 782 Tamils, 2013–2017. *Wald p* = multi-parameter Wald test *p*-value (one-tailed) for the test statistic *χ*^2^ and degrees of freedom d.f. (*H_Null_*: effects added in larger model over those in nested model are all equal to zero; *Wald p* < 0.05 is desirable). To clarify, consider, for example, the fully interacted SAOM where the *χ*^2^ test statistic for the multi-parameter Wald test of whether the effects added in Model 2 over those in Model 1 are all simultaneously equal to zero is 101.73 (d.f. = 20; *p* < 0.001). Thus, there is compelling evidence to support the addition of the effects in Model 2. *MHD* = joint Mahalanobis distance. *GOFp* = Monte Carlo MHD test *p*-value (*H_Null_*: Observed and simulated distribution are the *same*; MHD approaching zero and GOF*p* > 0.05 are desirable; one-tailed). In-degree range = 0–64. Out-degree range = 0–32. Geodesic distances range = 1–13 and infinity. MHD should only be compared across GOF tests for the same distribution. Recall that effects in SAOMs are summaries of features of local networks. Accordingly, it is unsurprising that the formal goodness-of-fit tests indicate that, relative to Model 1, Model 2 and Model 3 reproduce the distribution of differences in the gender-specific averages of the ego-net features under consideration.

multi-parameter Wald test	baseline model (1)			fully interacted model (2)			social constraints model (3)		
	*χ*^2^	d.f.	Wald *p*	*χ*^2^	d.f.	Wald *p*	*χ*^2^	d.f.	Wald *p*
model 2 versus model 1	—	—	—	90.05	17	<0.001	—	—	—
model 3 versus model 2	—	—	—	—	—	—	65.46	8	<0.001
									
***Lospinoso & Snijders*** **[83]** ***GOF test***	**MHD**	**GOFp**		**MHD**	**GOFp**		**MHD**	**GOFp**	
in-degree distribution	74.199	0.041	—	66.434	0.046	—	63.043	0.057	
out-degree distribution	8.238	0.992	—	8.927	0.985	—	8.560	0.990	
distribution of geodesic distances	30.149	0.039	—	26.823	0.031	—	25.692	0.042	
triad census	15.478	0.409	—	9.200	0.857	—	8.484	0.897	
ego-network statistics × gender	74.624	0.000	—	0.001	1.000	—	0.006	1.000	

**Figure 6.**
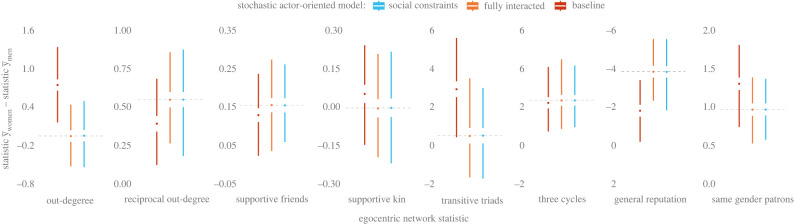
No substantial differences in the structure of men's and women's egocentric networks. Each bullet point (i.e. the middle of each Edward-Tufte-style box plot) indicates the median difference in the gender-specific averages of an ego-net statistic across networks simulated at the end of the observation period (2017). The mean of an ego-net statistic for men is subtracted from the mean of that statistic for women. Positive values indicate that the ego-nets of women have, on average, more of a statistic relative to the ego-nets of men. For example, *Out-degree* indicates that, in 2017, the observed difference (dashed horizontal line) between the average size of men's out-ego-nets and women's out-ego-nets is roughly zero. However, the median difference in the average across the 10 000 networks simulated under the Baseline model is roughly 0.6. The gaps immediately above and below the bullet points indicate the interquartile range (i.e. the values between the 75th and the 25th percentile). And the lines denote the whiskers—where the terminus of each whisker indicates the maximum/minimum of the distribution of values. Statistics capture ego's number of supportive alters that: (i) do not rely on ego (*Out-degree*); (ii) also rely on ego (*Reciprocal Out-degree*); (iii) are considered a friend by ego (*Supportive Friends*); (iv) are kin (*Supportive Kin*); and (v) are the same gender as ego (*Same Gender Patrons*). *Transitive Triads* and *Three Cycles* are, respectively, counts of the number of out-bound two-paths [*i* → *h* → *j*] and the number of in-bound two-paths [*i* ← *h* ← *j*] closed by ego's outgoing ties. *General Reputation* is the sum of the absolute value of the differences between the Z-score of the square root of the generation-reputation nominations of ego and each of their alters. That is, *General Reputation* is given by $\underset{j}{\sum }x_{ij}\left|{\sqrt{\mathrm{General}\;\mathrm{Reputation}}}_{i}-{\sqrt{\mathrm{General}\;\mathrm{Reputation}}}_{j}\right|$, where *x_ij_* = 1 if *i* seeks help from *j*. This sum is averaged across all men and all women and then compared. We are grateful to anonymous reviewer 1 for proposing this comparison of ego-net statistics.

The differences in the ego-net statistics (figure 6) provide little evidence to support our hypotheses. There appears to be no substantial difference between the average size of men's and women's ego-nets—whether measured by out-degree (H1), reciprocal out-degree (H2), number of supportive kin (H3), number of supportive friends (H4) or number of same gender patrons (H7) (cf. Figure 1). And these comparisons do not point to substantial divergence in the average number of out-bound two-paths [*i* → *h* → *j*] or the number of in-bound two-paths [*i* ← *h* ← *j*] closed by women's and men's outgoing ties (H5a [Transitive Triads]; H5b [Three Cycles]) (cf. Figure 1). However, there is support for H6 as, on average, women's ego-nets have a lower level of difference between the general reputations of ego and alter (i.e. men have, on average, alters with a greater difference in social standing relative to the global mean of $\sqrt{\mathrm{General}\;\mathrm{Reputation}}$).

Returning to figure 5, note well the *z*-axes (i.e. the vertical axes) for our linear combinations which indicate modest differences in the overall value of the evaluation function for men and women for the set of effects associated with each of our hypotheses across our artificial scenarios for a ministep, save the linear combinations related to H5. In this respect, a lack of substantial divergence in the observed structure of men's and women's ego-nets is not surprising.

## 4. Discussion

Here we have investigated the temporal evolution of social support in relation to gender using data on named sources of aid from nearly all adults in two Tamil villages. Our dynamic network models indicate that men and women differ in the strategies they use to informally access aid. However, these differences are not wholly consistent with predictions about male and female sociality found in prior evolutionary work.

Specifically, our results indicate that, relative to a man who is otherwise identical, a woman is more inclined to create and maintain a supportive bond in general (H1) and in relation to most of the cooperative mechanisms considered here—namely, reciprocity (H2), kin bias (H3), friend bias (H4), cyclic closure (H6), status-based assortativity (H6), and gender-based assortativity (H7). This finding broadly conforms to evolutionary predictions. However, these relational preferences do not apply to all women equally as structural position appears to heavily shape whether creating and maintaining a helpful tie is relatively more or less attractive to a man or a woman. And, generally speaking, women's greater inclination to create and maintain supportive bonds appears to only hold under moderate-to-high levels of ego activity (i.e. larger out-degrees)—particularly when women petition men (H1-H4, H6) or women (H7) with moderate-to-high levels of popularity (i.e. larger in-degrees). Only our hypothesis related to polyadic groups (H5) is clearly supported as men are markedly more inclined to create and maintain helpful connections embedded within transitive (but not cyclic) groups irrespective of structural position.

Still, the observed gender-based differences in the micro-level dynamics of help seeking are modest (figure 5, *z*-axes). Indeed, these differences do not appear to translate to women having, on average, considerably more supportive ties in general (H1), with mutual contacts (H2), with kin, (H3), or with friends (H4), or considerably fewer supportive ties with same-gender peers (H7) (figure 6). Nor does a greater attractiveness to transitive closure (figure 5) appear to translate to men being embedded in considerably more transitive groups (H5) (figure 6), although women are embedded in more cyclic groups (H5).

Ultimately, our findings are most consistent with the notion that males and females are similarly social but channel their sociality differently [18]. However, these differences appear to be small—and our findings are wholly inconsistent with the idea that help seeking on the part of females is somehow ‘rare’, ‘dyadic’, ‘parochial’, or ‘less group-based’ relative to males or that females are limited to the petitioning of friends and kin [17,18,23,47].

Nevertheless, our study has several important limitations. First, our findings are correlational and necessarily preliminary as they come from a single observational case study of two villages. Second, our data and the nature of the SAOM itself limit us to a discussion of proximate-level, mechanistic explanations [58,59,84,85] of social support. That is to say, our study only speaks to *how* social support comes about vis-à-vis network configurations (e.g. cyclic triads) taken, *a priori*, as representative of theorized social dynamics [86]. Thus, our results are silent on the ultimate [84,85] benefits of aid (i.e. what is the biological utility of social support vis-à-vis network dynamics?). That said, given our findings around polyadic groups (H5a), we note interesting new research on the adaptive value of transitivity, namely its ability to modulate conflict (see [48,87–91] as well as Ilany *et al*. [92,93]). Third, we have analysed self-reported sources of aid measured with slightly different questions between the two waves of data collection (electronic supplementary material, *Network Measurement*) as opposed to observed resource flows (e.g. see the experimental design of Smith *et al*. [94]). Fourth, despite our data on familial links between the residents of Alakāpuram and Tenpaṭṭi having good coverage, our information on very-distant relatives is less complete (see the electronic supplementary material of Ready & Power [63, pp. 2–3 and 5–7]), leading to mild truncation of our measures of relatedness (electronic supplementary material, *Kinship Measurement*). Last, we only analyse relationships within our study villages. And, despite Alakāpuram and Tenpaṭṭi being large, our inability to model cooperation beyond the two adjacent communities is not ideal as help need not be local [95].

Still, the most important shortcoming of our study is its temporal design. While we add to human evolutionary science by providing a rare longitudinal analysis of a social support network that spans nearly all men and women in two sizeable populations, our reliance on two waves of data prevents us from examining the intricate dynamics of help seeking. For instance, the adjacency matrices to which we fitted our SAOMs exhibit a relatively low level of similarity between waves (see electronic supplementary material, table S3 on ‘Jaccard similarity’). And this instability of the adjacency matrices makes us wary of disaggregating SAOM effects to separately explore: (i) the association between gender and the creation of wholly new ties; and (ii) the association between gender and the maintenance of old connections [31]. Similarly, as we only have two waves of data, we cannot explore temporal heterogeneity in the network dynamics evidenced by our models (e.g. see Redhead & von Rueden [27,28]). This precludes us from examining if, for instance, the parameter estimate for *Reciprocity* is consistently positive over time.

Despite these limitations, there are promising avenues for future work seeking to replicate and extend our analysis. And we strongly suggest that evolutionary scientists reorient their attention away from sex and egocentric-network size to instead use generative models to explore the interplay between sex and cooperative mechanisms within population-spanning networks given their contextualization of relational behaviour (see Abbott [96] on networks, time, and space). Along this line, in two papers introducing SAOMs to the human evolutionary sciences, Redhead & von Rueden [27,28] analyse social support networks of Tsimané males over 8 years, documenting similar dynamics to what we have observed (e.g. transitivity). However, as noted in our introduction, there may be important differences in cooperation within and between the two sexes [21,23]. Thus, future research should explore whether the strategies that males and females use to access and provide help differ based on the sex of aid sources and aid targets. For example, are males more inclined to create and maintain aid relationships embedded in transitive groups in general ([*i_M_* → *h_M_*_or *F*_ → *j_M_*_or *F*_ ← *i_M_*])? When these bonds are only with males ([*i_M_* → *h_M_* → *j_M_* ← *i_M_*])? Or when these bonds are with females ([*i_M_* → *h_F_* → *j_F_* ← *i_M_*])?

Additionally, future work on sex and sociality should contrast the dynamics of whole networks of distinct types of aid—not simply whether one network (e.g. friendship) is associated with another (e.g. medical advice) via link superimposition (figure 3) or mixed transitivity (e.g. see Redhead & von Rueden [28]). Indeed, certain forms of help may simply be more relevant to one of the two sexes given differences between males and females with respect to their reproductive and economic strategies. The most obvious example is the importance of childcare for females given the requisite physical and time investments necessitated by maternity and the rearing of adolescents. Accordingly, possible sex-based differences in the dynamics governing alloparenting networks [10,97–100] could differ from those that may characterize, for example, networks of agriculture-related advice [12] and networks of friendship [22,29,31,43,47,101,102].

Finally, barring experiments [94], large-scale meta-analyses of the dynamics of many complete, inter-individual networks (e.g. see [73,103,104]) sampled from heterogenous settings are sorely needed as they allow for conclusions more definitive than what we can provide here. We acknowledge that sociometric censuses and ethnographic fieldwork to collect longitudinal data are difficult [105,106]. However, as we discuss in our electronic supplementary material (*Sociological Determinants of Social Ties*), prevailing socioecological conditions are likely to shape human social networks.

Recall that our study villages are both patrilocal and patrilineal. As a result, they are male biased in practice. And, in recent years, Tamil Nadu has gone through a number of economic and demographic changes (e.g. increased market integration, higher levels of school and university attendance, and lower fertility) that have altered traditional kinship and family structure (see the electronic supplementary material of Ready & Power [63, pp. 1–3] for a discussion). Based on prior cross-sectional analyses of our 2013 and 2017 data [52,63], male bias does not seem to depress women's number of supportive bonds due to immigrant women counterbalancing a lack of consanguineal kin with aid from affines [63]. Nor does male bias prevent the women in our study villages from occupying influential positions in the local social order [52], even if they are unlikely to be widely regarded as ‘influential’ (i.e. prominent, possessing authority) by other residents compared to men [52]. These social dynamics—in addition to the mixed-subsistence economy (smallholding plus wage-labour), the level of market integration, and the patrilocal and patrilineal context—naturally raise the question of whether our results hold for social support networks measured in societies with different norms around work, schooling, outside contact, post-marital residence, and descent. Indeed, there is evidence to suggest that males' and females’ accrual of supportive social bonds unfolds differently under matriliny compared to patriliny (e.g. see Mattison *et al*. [16,107] and Seabright *et al*. [108], both in this issue, in addition to Macfarlan *et al*. [19]).

## Data Availability

All R code required to replicate our analyses is available via GitHub: https://github.com/cohensimpson/gendernet_PhilTransB. As they contain sensitive data (e.g. geolocation), we have not posted the data files in our GitHub repository alongside our R code. To access these files, please contact the second author and data controller E.A.P. (e.a.power@lse.ac.uk) to sign an ethics and data sharing agreement, cc'ing the first author C.R.S. (c.r.simpson@lse.ac.uk). The electronic supplementary material for our paper appears on Figshare [109].
